# Proteomic and miRNA profiling of radon-induced skin damage in mice: FASN regulated by miRNAs

**DOI:** 10.1093/jrr/rrac037

**Published:** 2022-07-05

**Authors:** Wei Mo, Wanglei Xu, Min Hong, Tingyi Yang, Yuhong Shi, Yang Jiao, Jihua Nie, Fengmei Cui, Jianping Cao, Shuyu Zhang

**Affiliations:** School of Radiation Medicine and Protection, State Key Laboratory of Radiation Medicine and Protection, Soochow University, Suzhou 215123, China; School of Radiation Medicine and Protection, State Key Laboratory of Radiation Medicine and Protection, Soochow University, Suzhou 215123, China; School of Radiation Medicine and Protection, State Key Laboratory of Radiation Medicine and Protection, Soochow University, Suzhou 215123, China; West China School of Basic Medical Sciences & Forensic Medicine, Sichuan University, Chengdu 610041, China; Second Affiliated Hospital of Chengdu Medical College, China National Nuclear Corporation 416 Hospital, Chengdu 610051, China; School of Radiation Medicine and Protection, State Key Laboratory of Radiation Medicine and Protection, Soochow University, Suzhou 215123, China; School of Radiation Medicine and Protection, State Key Laboratory of Radiation Medicine and Protection, Soochow University, Suzhou 215123, China; School of Radiation Medicine and Protection, State Key Laboratory of Radiation Medicine and Protection, Soochow University, Suzhou 215123, China; School of Radiation Medicine and Protection, State Key Laboratory of Radiation Medicine and Protection, Soochow University, Suzhou 215123, China; West China School of Basic Medical Sciences & Forensic Medicine, Sichuan University, Chengdu 610041, China; Second Affiliated Hospital of Chengdu Medical College, China National Nuclear Corporation 416 Hospital, Chengdu 610051, China; West China Second University Hospital, Sichuan University, Chengdu 610041, China

**Keywords:** Radon, skin, protein profiling, miRNA profiling, fatty acid synthase (FASN)

## Abstract

Radon is a naturally occurring radioactive gas and considered as a serious carcinogen to humans. Continuous radioactive decay of this gas emits high-energy alpha particles. Long-term radon exposure induces oxidative stress and inflammatory response, which results in chronic lung diseases. However, biological effects after radon exposure in other organs have been rarely reported. As the outermost organ of the human body, the skin suffers from environmental damage to agents such as air pollution. Epidemiological studies indicated that areas with high level of radon had a high incidence of skin cancer. However, whether radon exposure induces skin damage has not been reported yet. In this study, we established a radon-exposed mouse model and found that radon exposure affected the structure of skin tissues, which was manifested by inflammatory cell infiltration and skin atrophy. Using proteomic approach, we found 45 preferentially expressed proteins in 60 Working Level Months (WLM) group and 314 preferentially expressed proteins in 120 WLM group from radon-exposed skin tissues. Through microRNA (miRNA) sequencing profiling analysis, 57 dysregulated miRNAs were screened between the control and radon-treated mouse skin. By integrating the dysregulated proteins and miRNAs, radon-induced fatty acid synthase (FASN) was investigated in greater detail. Results showed that FASN was regulated by miR-206-3p and miR-378a-3p and involved in the pathogenesis of radon-induced skin damage. Overexpression of FASN inhibited the proliferation, and induced in WS1 cells. Our present findings illustrate the molecular change during radon-induced skin damage and the potential role of FASN during this process.

## INTRODUCTION

Radon is a ubiquitous radioactive gas that is responsible for approximately half of the human annual background radiation exposure globally and is the decay product of uranium [1, 2]. Continuous radioactive decay of this gas emits high-energy alpha particles that may interact with biological tissues and macro-molecules. Similar to other ionizing radiation, radon and its decay products are mutagenic, as they could lead to single and double-strand DNA breaks, pyrimidine dimer formations, intra-and inter-chromosomal aberrations sister chromatid exchange micronuclei formation and eventually genomic instability [3]. It is indicated that alpha-particle exposure induces mainly unstable complex chromosome aberrations which do not contribute to radiation-associated cytogenetic risk [4]. Through many epidemiological studies regarding occupational exposure among miners and residential exposure among the general population, radon has been scientifically proven to cause lung cancer and radon exposure is the second most common cause of lung cancer after cigarette smoking [5–7]. Additionally, cumulative radon exposure was associated with higher risk of ER negative breast cancer [3]. However, biological effects after radon exposure in other organs have been rarely reported.

The skin, as the outermost organ of the human body, functions as an effective physical and immunological barrier against external stimulation. At the same time, the skin suffers from environmental damage to agents such as air pollution and cigarette smoking. It is the exposed organ of radon and radon progeny. Annual dose to the skin at 200 Bq/m^3^ is estimated to be 25 mSv [8]. As such, the skin receives the second-highest dose after the respiratory tract; Environmental radon is also relevant for skin exposure because radon attaches to aerosol particles in the air, which adhere to the human skin via electrostatic attraction [9]. Subsequently, radon progeny plate out on skin and give rise to exposure of the superficial epidermis from alpha emitters Po-218 (7.7 MeV, range approximately 66 microm) and Po-214 (6 MeV, range approximately 44 microm). Dose rates from β/γ emitters Pb-214 and Bi-214 are low and only predominate at depths in excess of the α range [10]. Radon exposure has been suggested as the cause of increased risk of skin malignancy. The epidemiological survey of high background radon areas in Europe, Evidence from countries such as Switzerland and Denmark, reveals that long-term exposure to high background radon can increase the development of skin basal cell carcinoma [1, 11]. However, direct experimental evidence in radon-induced biological effect in skin remains unreported.

In general, most biological functions are performed and regulated by proteins and noncoding Ribonucleic Acids (RNAs), including microRNAs (miRNAs). Systematic analysis of the whole proteome may extend our understanding of the molecular pathogenesis of this injury. It may also provide precious comprehensive information about the molecular basis of radon-induced skin damage and the course of the damage.

MiRNAs are an abundant class of ~22 nucleotide (nt) long evolutionarily conserved endogenous non-coding small RNA molecules that are derived from ~70 nt long stem-loop precursors (pre-miRNAs) [12]. Lines of evidence suggest that miRNAs play important roles in a wide range of physiological and pathological processes, including cellular proliferation, differentiation, development and immune response [13, 14]. About 60% of mammalian genes are predicted to be regulated by miRNAs [15]. miRNAs are key post-transcriptional regulators of gene expression via either translational repression or mRNA degradation in mammals [16]. Thus, identification of the miRNAs involved in radon-induced skin damage may provide new insights into its progression.

The protein and miRNA profiles of radon-exposed skin tissues have not been analyzed, and there is currently little information published on the underlying mechanism. Extensive investigation of the molecular etiology as well as the treatment of radon-induced skin injury is warranted. In this study, we established a radon-exposed mouse model and found that radon exposure can affect the structure of skin tissue, which is manifested by inflammatory cell infiltration and skin atrophy. We further investigated the protein and miRNA profiles of radon-exposed mouse skin. Radon-induced fatty acid synthase (FASN) expression and its functional significance was characterized.

## MATERIALS AND METHODS

### Reagents

The FASN overexpression plasmid was constructed by PPL Biotech (Nanjing, China) and confirmed by sequencing. The miRNA mimics and miRNA Inhibitor were synthesized by GenePharma (Suzhou, China). The sequences synthesized RNAs are shown in Supplementary Table 1. DAPI were purchased from Sigma Chemical (St. Louis, MO).

### Animals and radon exposure

Female BALB/c mice (8 weeks old, weighing 18–22 g) were purchased from the Shanghai SLAC Laboratory Animal Co, Ltd (Shanghai, China). The animals were housed and maintained in a 12-hour light/dark cycle and had free access to food and water. The mice were placed in a multi-functional radon chamber with 10^5^ Bq/m^3^ of radon and its daughters from radium decay. Experimental mice (three mice/group) were exposed to radon for 10 h/day, 6 days/week and up to 189 h/377 h/755 h, which were equivalent to cumulative doses of 30/60/120 Working Level Months (WLM), respectively. The control mice were housed in a room with a background concentration of radon at 80 Bq/m^3^. The radon concentration was constantly maintained and continuously measured by emanometer (NT8260, Jiangsu Suhe Instrument co. LTD) [17–19]. Protocols for experiments involving animals were approved by the Animal Experimentation Ethics Committee at Soochow University (Suzhou, China).

### Hematoxylin and eosin staining

The skin tissues were fixed in 10% neutral-buffered formalin and embedded in paraffin. Three-micrometer paraffin sections were deparaffinized and heat-treated using citrate buffer (pH = 6.0) for 7 min following an epitope retrieval protocol. The sections were stained using hematoxylin and eosin (H&E) (ZSGB-Bio, Beijing, China). All slides were examined under a microscope and photographed. Representative images were randomly selected from each sample.

### Immunofluorescence assay

Cryosections and cells were washed with phosphate-buffered saline (PBS), and blocked with 5% FBS/PBS at room temperature. Then antibodies against F4/80 (1:100, GeneTex, San Antonio, TX, USA), MPO (1:100, ImmunoWay, Plano, TX, USA), γ-H2AX (1:1000, Abcam, TX, USA), Caspase-3 (1:500, GeneTex, San Antonio, TX, USA) were incubated with slides overnight at 4°C, followed by Alexa Fluor 488-labeled goat-anti-rabbit or Alexa Fluor 555-labeled goat-anti-rabbit (Beyotime, Nantong, China) secondary antibody (1:1000) for 1 h at room temperature. To stain the nuclei 4′,6-diamidino-2-phenylindole (DAPI; Beyotime, Nantong, China) was used, and images were captured using a scanning laser confocal microscopy (Olympus, Tokyo, Japan). All antibodies in this experiment were diluted with PBS.

### Senescence-associated β-galactosidase (SA-β-gal) analysis

SA-β-gal in mouse skin tissues after radon irradiation was detected by a Senescence β-Galactosidase Staining Kit (Beyotime, Nantong, China), according to the instruction of manufacturer.

### Transmission electron microscopy

Skin tissues were fixed for 2 hours with 2.5% glutaraldehyde in 0.05 M sodium cacodylate buffer, pH 7.2 at room temperature, followed by 2 hours in 2% OsO4 in 0.1 M sodium cacodylate buffer and 18 hours in 1% aqueous uranyl acetate solution. After dehydration through an ethanol series, the specimens were embedded in Epon 812 and ultrathin sections were collected on copper grids. After being stained with uranyl acetate and lead citrate, the sections were examined using a Tecnai G2 Spirit BioTwin transmission electron microscope (FEI Company, Hillsboro, Oregon, USA).

### Cell culture and radon exposure

Human skin fibroblasts (WS1) were purchased from American Type Culture Collection (ATCC, Gaithersburg, MD, USA). The cells were maintained in Dulbecco’s modified Eagle’s medium (DMEM) supplemented with 10% fetal bovine serum (Gibco, Grand Land, NY, USA). The cells were cultured in an incubator at 37°C, with 5% CO_2_.

For radon exposure, approximately 1 × 10^5^ cells were seeded on a transwell chamber in culture plates (Costar, New York, NY, USA). The following day, the plates placed in a gas inhalation chamber (MED8170, Tianjin Hope Industry & Trade Co. Ltd, Tianjin, China). The gas chamber was connected to a multifunctional radon chamber purchased from Donghua University (Shanghai, China). Radon and its progeny were produced by a radium source and pumped into cell chambers using a pump machine. The medium on the upper chamber of the transwell plate was removed, and the cells were exposed to radon (20,000 Bq/m^3^) for 30 min, fresh medium was added and cultured for 3 days. Cells were subsequently exposed to radon again at the same concentration and duration [17]. Cells underwent five instances of repeated exposure. Control cells were cultured without radon exposure.

### Cell cycle analysis

WS1 cells were exposed with different dose radon for certain times or transfected with control vector (pcDNA3.1) or FASN expression vector. After 24 h, cells were harvested and fixed with 75% ethanol. Cells were resuspended in PBS, incubated at 37°C for 20 min and stained with propidium iodide (PI) (Beyotime, Nantong, China). Cell cycle distribution was then evaluated by flow cytometry (>10 000 cells per sample).

### Cell viability assay

The cell viability 3-(4,5-dimethylthiazol-2-yl)-2,5-diphenyl-2Htetrazolium bromide (MTT) assay was carried out in 96-well plates. The cell viability was detected 24 h after WS1 cells were exposed to radon.

### Measurement of cell apoptosis

Apoptosis was measured using PI/annexin V double staining (Beyotime, Nantong, China). The cells were harvested five generations after exposure to 0 or 120 WLM radon. Apoptotic fractions were measured using flowcytometry (Beckman, Fullerton, CA, USA).

### Western blotting analysis

The cells were washed twice with ice-cold PBS and directly lysed in 200 μl of cell lysis buffer. The lysates were boiled, centrifuged at 10000 rpm and then loaded onto a 6% SDS–PAGE gel. The samples were electrophoresed for 2 h and transferred onto transfer membranes. After being blocked with 5% BSA in PBS–Tween 20 for 1 h at room temperature, the membranes were blotted with FASN antibody (Abcam, Cambridge, MA, USA) or α-Tubulin primary antibodies (Beyotime, Nantong, China) at 1:1000 dilutions. The membranes were then incubated with the appropriate horseradish peroxidase-coupled secondary antibody at a 1:2000 dilution for 1 h at room temperature. After the membranes were washed with Tris-buffered saline–Tween 20, the blots were incubated with enhanced chemiluminescence (ECL) stable peroxide solution (Beyotime, Nantong, China). All blots were visualized using a FluoroChem MI imaging system (AlphaInnotech, Santa Clara, CA, USA) at room temperature.

### Statistical analysis

Data were expressed as mean ± standard error of the mean of at least three biological replicates. Student’s t test and one-way analysis of variance (ANOVA) were performed using GraphPad Prism (San Diego, CA). The statistical analyses were performed using SPSS version 16.0 statistical software (Chicago, IL USA). The differences were considered significant at *P* < 0.05. ^*^P < 0.05, ^*^^*^P < 0.01, compared to control group.

Other materials and methods are available in the Supplementary Materials and Methods.

## RESULTS

### Radon exposure resulted skin tissue damage in mice

**Fig. 1:**
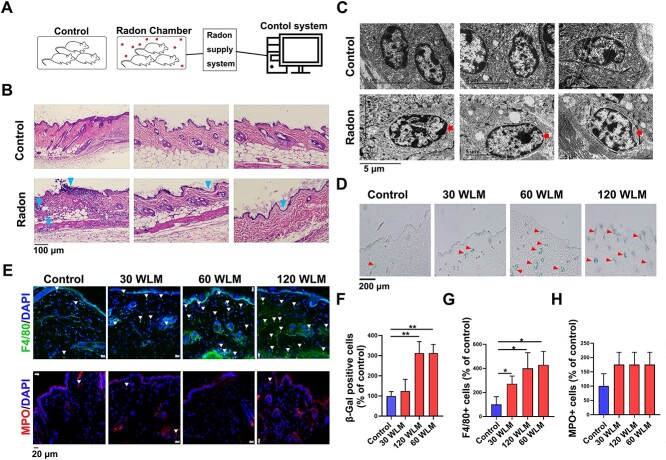
Radon induces skin morphological and physiological changes in mouse models. (A) Schematic diagram of radon exposure. (B) Representative H&E staining of skin tissues from radon irradiated and nonirradiated mice, magnification, ×200. (C) Electron microscopy analysis of mouse skin tissues after radon irradiated and nonirradiated. Red arrows represent swelling mitochondrion. (D) The SA-β-gal analysis in mouse skin exposed to radon. Red arrows represent β-gal positive cells. (E) The immunofluorescence staining for F4/80, MPO of radon-exposed mouse skin. Arrows represent F4/80^+^ and MPO^+^ cells. (F) The quantification of SA-ß gal, MPO (G) and F4/80 (H) staining.

The mice were placed in a multi-functional radon chamber with 10^5^ Bq/m^3^ of radon and its daughters from radium decay. Experimental mice were exposed to radon for 10 h/day, 6 days/week and up to 189 h/377 h/755 h, which were equivalent to cumulative doses of 30/60/120 WLM, respectively [17]. The control mice were housed in a room with a background concentration of radon at 80 Bq/m^3^(Fig. 1A). To investigate the effect of radon exposure on skin, mouse skin tissues were collected and observed by H&E staining. Results showed that in radon-exposed skin tissues, there was obvious swelling of the subcutaneous appendages and thinning in the epidermis of the skin tissue (Fig. 1B). Under electron microscope, abnormal nucleus and mitochondria were observed in skin tissue exposed to 120 WLM radon (Fig. 1C). Next, we investigated whether radon affected the cells’ aging in mouse skin tissues. As shown in Fig. 1D and F, radon exposure increased the positive rate of SA-β-gal staining in a dose-dependent manner. The abundance of neutrophils and macrophages in skin tissues was assessed by staining for the cell markers MPO and F4/80. While there was no difference in the density of MPO+ cells between the groups (Fig. 1H), F4/80+ cells were increased after radon exposure compared to the control (Fig. 1E and G). All the above results suggested that radon exposure affected the structure of skin tissues, which was manifested by cell aging, subcutaneous appendages and mitochondria swelling, inflammatory cell infiltration and skin atrophy.

### Radon exposure induced cell apoptosis in human skin fibroblasts

**Fig. 2:**
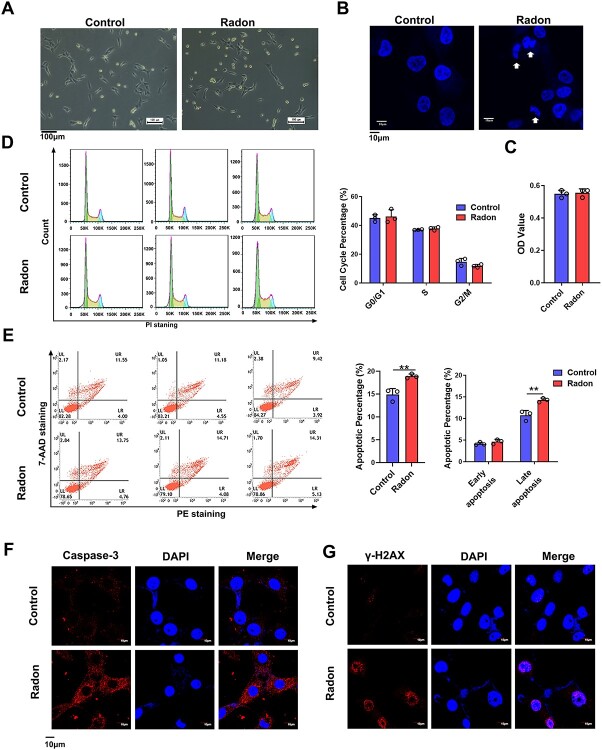
Radon exposure induced cell apoptosis in skin fibroblasts. (A) Images of normal WS1 and radon-exposed WS1 cells. (B) Representative nucleus images of normal WS1 and radon-exposed WS1 cells. Nuclei were counterstained with DAPI. (C) Cell proliferation ability of WS1 cells exposed to radon. (D) Cell cycle analysis in WS1 cells exposed to radon. (E) Cell apoptosis rate of WS1 cells measured by flow cytometry. Early apoptotic cells (Annexin V-PE^+^/PI^+^; shown as LR); Late apoptotic cells (Annexin V-PE^+^/PI^+^; shown as UR); UL, upper left; UR, upper right; LL, left lower; LR, lower right; (F) and (G) The distribution of Caspase-3 and γ-H2AX in WS1 cells detected by Immunofluorescence experiment.

To investigate the effect of radon exposure on WS1 cells, the cells were exposed to radon (20 000 Bq/m3) for 30 min, a fresh medium was added and cultured for 3 days. Cells subsequently exposed to radon again at the same concentration and duration [17]. This process was repeated five times. Control cells were cultured without radon exposure. Under microscope, there was no abnormal in morphology of WS1 after radon exposure, compared with the normal cells (Fig. 2A). However, the nuclear fragmentation (Fig. 2B) and the presence of γ-H2AX indicative of DNA double strand breaks was detected in the radon-exposed WS1 cells under confocal microscope (Fig. 2G). We then examined if radon affects the biological behavior of skin fibroblasts WS1 cells. Results indicated that the proliferation of WS1 exposed to radon did not show significant differences (Fig. 2C). Cell cycle analysis showed that there was no distinct effect on cell cycle of WS1 after radon exposure (Fig. 2D). Comparatively, radon-exposed WS1 cells showed a significant increase in apoptotic cells, especially late apoptosis cells (Annexin V-PE^+^/PI^+^, Fig. 2E). Radon exposure increased the total apoptotic percentage of from 14.87 ± 1.09 up to 18.91 ± 0.39. The late apoptosis was significantly increased from 10.72 ± 0.93 up to 14.26 ± 0.39 after radon exposure, respectively. Moreover, the expression of caspase-3 was detected in the radon-exposed WS1 cells (Fig. 2F). These results showed that radon exposure can increase the rate of cell apoptosis and change the morphology of the cell nucleus, resulting in cell pyknosis, fragmentation and DNA double strand breaks.

**Fig. 3:**
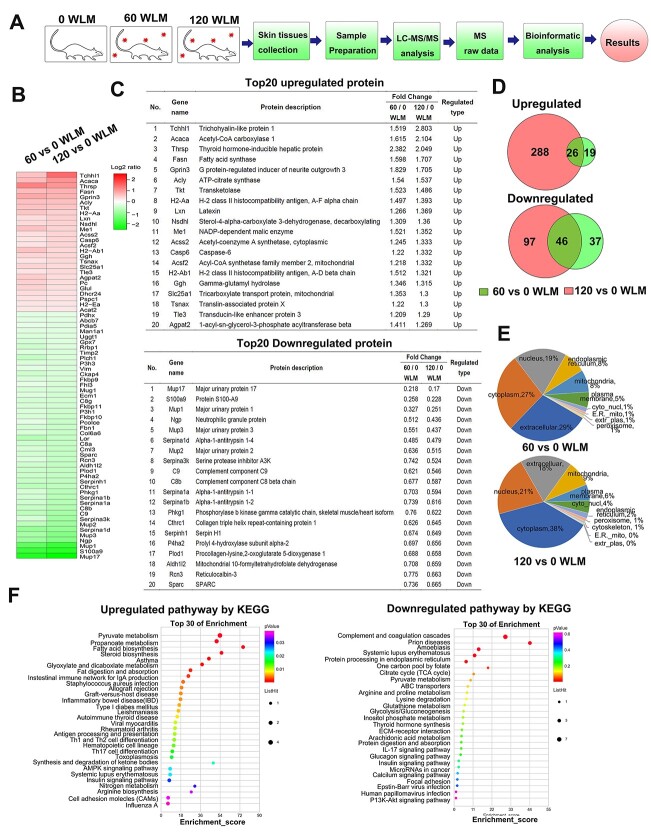
Proteomic analysis of mouse skin tissues subjected to 60 and 120 WLM radon irradiation. (A) Experimental design of the proteomic analysis. (B) The heatmap of significant differently expressed proteins in both 60 and 120 WLM radon-exposed skin tissues. Significant defined as fold change (FC) >1.2 and *P* value <0.05. (C) The list of top 20 dysregulated proteins in both 60 and 120 WLM radon irradiated groups. (D) Venn diagram significant differently expressed proteins in 60 vs 0 WLM and 120 vs 0 WLM radon irradiated skin tissue samples. (E) Subcellular locations of up-regulated and down-regulated proteins (60 or 120 WLM radon-exposed skin tissues vs control skin tissues group). (F) The KEGG pathway enrichment analysis of up-regulated and down-regulated proteins in both 60 and 120 WLM radon irradiated groups.

### Multiple proteins were dysregulated in mouse skin tissues after radon exposure

To investigate the molecular mechanism of radon-induced skin tissue damage in mice, the proteomic profile of mouse skin tissues exposed to radon in 0, 60 and 120 WLM was investigated by tandem mass tag (TMT)-based protein quantification (Fig. 3A). A total of 4115 proteins were successfully identified, with 45 significantly up-regulated and 83 significantly down-regulated proteins identified in 60 WLM. In addition, with 314 significantly up-regulated and 143 significantly down-regulated proteins identified in 120 WLM. There are 72 common dysregulated proteins (26 up-regulated and 46 down-regulated) were identified in both 60 and 120 groups (Fig. 3B and D). The list of top 20 dysregulated proteins is shown in Fig. 3C. The up-regulated proteins in both 60 and 120 WLM included thyroid hormone-inducible hepatic protein (THRSP), acetyl-CoA carboxylase 1 (ACACA), FASN, ATP-citrate synthase (ACLY). The down-regulated proteins included major urinary protein 17 (Mup17), protein S100-A9 (S100a9), major urinary protein 1 (Mup1).

To better understand the functions and features of the identified proteins, we used the Gene Ontology (GO) annotation of subcellular localization in the enrichment analysis. The proportion of differentially expressed proteins representing each subcellular location is summarized in Fig. 3E. Among the dysregulated proteins in 60 WLM, 29% were extracellular proteins, 27% were cytoplasm proteins and 19% were nuclear proteins. However, 38% were cytoplasm proteins, 21% were nuclear proteins and 18% were extracellular proteins, among the dysregulated proteins in 120 WLM.

The Kyoto Encyclopedia of Genes and Genomics (KEGG)-based pathway enrichment analysis revealed that fatty acid (FA) biosynthesis, pyruvate metabolism and propanoate metabolism pathways were significantly up-regulated, whereas complement and coagulation cascades was the most significantly down-regulated pathway after radon exposure (Fig. 3F). FAs are essential constituents of all biological membrane lipids, and are important substrates for energy metabolism. There are two sources of FAs for animal metabolism, exogenously-derived (dietary) FAs and endogenously-synthesized FAs. The biosynthesis of the latter is catalyzed by FASN. FASN synthesizes long-chain FAs by using acetyl-CoA as a primer, malonyl-CoA as a two-carbon donor and NADPH as a reducing equivalent [18]. In this study, FASN was significantly up-regulated post radon exposure. These results suggested that FASN may be involved in radon-induced skin damage.

### Radon modulates the expression of miRNAs in skin tissues

**Fig. 4:**
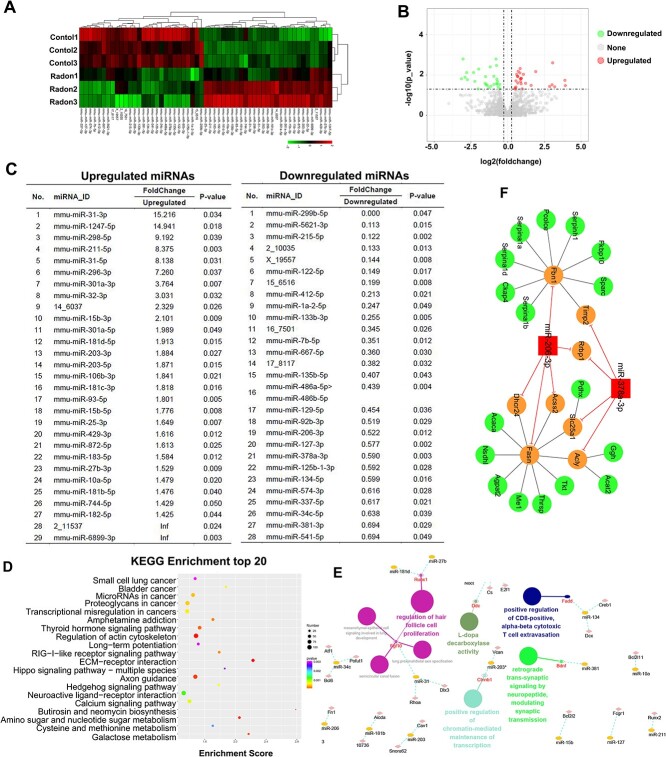
miRNA expression profile of skin tissues after radon irradiation. (A) and (B) Heatmap and volcano plot of the dysregulated miRNAs between the two groups (*n* = 3). Expression levels are reflected by color change. Red denotes higher expression, whereas green denotes lower expression. (C) Differentially expressed miRNAs in radon irradiated skin tissues of mice. (D) The KEGG pathway enrichment of the dysregulated miRNAs from mouse skin tissues in response to radon. (E) GO analysis of the dysregulated miRNAs were performed by ClueGO. (F) The miRNA regulated PPIN between dysregulated miRNAs and proteins. In this network, red node denotes the miRNA, yellow nodes denote miRNA directly targeted genes, and green nodes denote genes connected with target genes. The red lines represent a negative regulatory relationship initiated by miRNAs. The black lines represent interactions between protein and protein.

To search for miRNAs that may be involved in the progression of radon-induced skin damage, mouse skin tissues exposed to 0 or 120 WLM radon were screened for differentially expressed miRNAs by miRNA sequencing analysis. Overall, 1370 miRNAs were analyzed, of which 57 (4.16%) miRNAs were significantly dysregulated (29 up-regulated [50.88%] and 28 [49.12%] down-regulated) in radon exposure skin tissues (false discovery rate *P* < 0.05). Hierarchical clustering (Fig. 4A) showed clear separation between normal and radon groups, although there was heterogeneity within the individual clusters, possibly reflecting latent phenotypic differences between the samples. Up-regulated (*P* < 0.05; red circles) and down-regulated (*P* < 0.05; green circles) miRNAs are shown in Fig. 4B. The full list of dysregulated miRNAs can be found in Fig. 4C.

### Functional annotation of differentially expressed miRNAs

The KEGG pathway analysis was performed to investigate pathways that possibly involved in radon-induced skin damage. The enriched pathways included regulation of actin cytoskeleton, axon guidance, neuroactive ligand−receptor interaction and proteoglycans in cancer (Fig. 4D). Then, GO analysis of the dysregulated miRNAs was performed by ClueGO, the result was showed in Fig 4E. The miRNA regulated protein–protein interaction network (PPIN) between dysregulated miRNAs and proteins are shown in Fig. 4F. These results suggested that these miRNAs were likely to be involved in radon-induced skin damage.

### FASN is regulated by miR-378a-3p and miR-206-3p

**Fig. 5:**
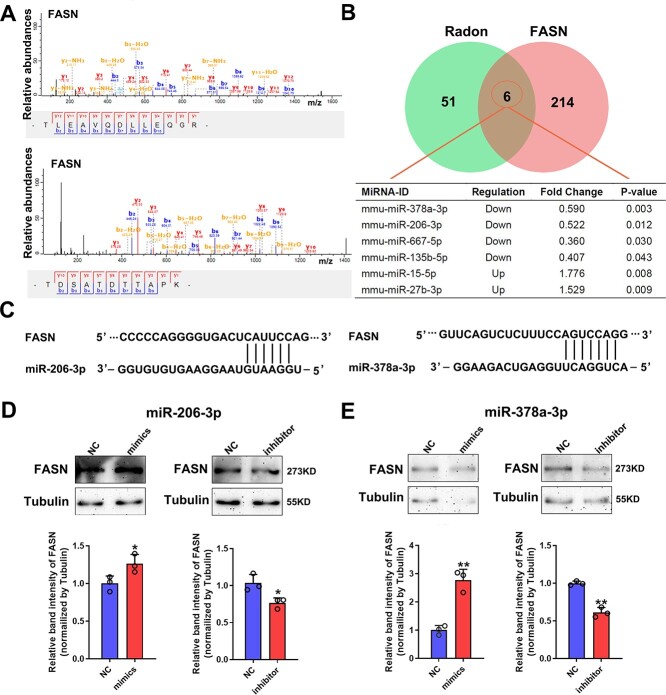
FASN is regulated by miR-378a-3p and miR-206-3p. (A) Mass spectrometric identification of FASN. (B) The intersection of the target miRNAs of FASN and miRNAs dysregulated in radon-exposed skin tissues. (C) Bioinformatics algorithm (TargetScan) was used to explore the possible interactions between miRNAs and proteins that had been altered by radon. (D) Protein levels of FASN in WS1 cells detected by Western blotting analysis post miR-206-3p mimics and inhibitor treatment. (E) Protein levels of FASN in WS1 cells detected by Western blotting post miR-378a-3p mimics or inhibitor treatment.

The proteomic profile showed that FASN was up-regulated in mouse skin tissues post radon exposure (Fig. 4C). FASN functions a large multi-enzyme complex and its monomeric protein size is ~270 kDa. FASN involves several signaling pathways that regulate metabolism, proliferation and survival in cells [21]. Mass spectrometric identification of FASN was showed in Fig. 5A. Bioinformatics algorithm (TargetScan) was used to explore the possible interactions between miRNAs and FASN proteins that had been altered by radon. We found 220 target miRNAs of FASN, of which six miRNAs were dysregulated in radon-exposed skin tissues (Fig. 5B). The results of bioinformatics analysis showed that miR-206-3p and miR-378a-3p, which were significantly down-regulated, might have a direct regulatory effect on FASN (Fig. 5C). Western blotting analysis indicated that the level of FASN protein was increased by transfection of miR-206-3p and miR-378a-3p mimics, whereas FASN expression was reduced by the transfection of miR-206-3p and miR-378a-3p inhibitors (Figs 5D and E). This result indicated that both miR-206-3p and miR-378a-3p positively regulated the expression of FASN.

### Overexpression of FASN affects the biological functions of skin fibroblasts

**Fig. 6:**
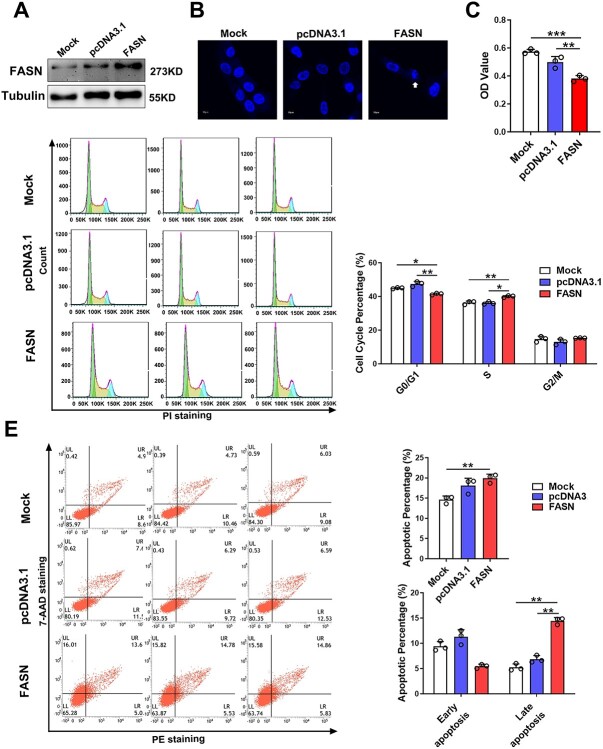
Overexpression of FASN affects the nucleus morphology and biological functions of human skin fibroblasts. (A) Western blotting analysis to detect FASN overexpression in WS1 cells. (B) Representative nucleus images of WS1 cells transfected with the FASN overexpression vector or control vector. Nuclei were counterstained with DAPI. (C) Cell proliferation of WS1 cells transfected with the FASN overexpression vector or control vector. (D) and (E) cell cycle and apoptosis analysis of WS1 cells transfected with the FASN overexpression vector or control vector.

We then explored the role of FASN in WS1 proliferation and apoptosis. Western blot assay showed that the level of FASN was increased after transfecting with the FASN expression vector (Fig. 6A). The nucleus morphology of WS1 were destroyed after overexpressing of FASN (Fig. 6B). As showed in Fig. 6C, overexpression of FASN suppressed the proliferation of WS1 cells. Cell cycle analysis showed that the number of the S phase of WS1 cells was increased after overexpression of FASN (Fig. 6D). Moreover, overexpression of FASN aggravated the apoptosis of WS1 cells, especially late apoptosis (Fig. 6E). These results suggested that overexpression of FASN affected the nucleus morphology and biological functions of WS1 cells.

## DISCUSSION

Radon is considered a human carcinogen by the International Agency for Research on Cancer (IARC). Long-term radon exposure can cause oxidative stress, inflammatory response and result in chronic lung diseases including pulmonary fibrosis and lung cancer. Since inhalation is the primary route of radon exposure, much of the current studies for radon focuses on lung cancer as the main health outcome. Additionally, cumulative radon exposure was associated with higher risk of ER negative breast cancer. The P38 MAPK signaling and phosphocholine synthesis gene sets were significantly enriched in ER negative breast tumor and ER negative normal-adjacent samples with higher cumulative radon exposure [3]. As the outermost organ of the human body, the skin suffers from environmental damage to agents such as air pollution and cigarette smoking. Epidemiological studies indicated that areas with high level of radon had a high incidence of skin cancer [1, 11]. However, whether radon exposure-induced skin damage has not been extensively reported. To our knowledge, this is the first observation that radon exposure induced cutaneous damage in a mouse model, which illustrated an unappreciated phenotype of radon. The changes in protein and miRNA expression in response to radon irradiation provided insight into the molecular pathogenesis of radon-induced skin damage.

As showed in proteomic analysis, there are 72 common dysregulated proteins (26 up-regulated and 46 down-regulated) were identified in both 60 and 120 WLM groups. The up-regulated and down-regulated proteins are likely responsible for the effect of radon-induced skin damage. The down-regulated proteins included Mup17, S100a9 and Mup1, etc. Whereas THRSP, ACACA, FASN, ACLY were up-regulated. These up-regulated genes are related to FA synthesis. FAs are essential constituents of all biological membrane lipids, and are important substrates for energy metabolism. There are two sources of FAs for animal metabolism, exogenously-derived (dietary) FAs and endogenously-synthesized FAs. The biosynthesis of the latter is catalyzed by FASN. It comprises six separate enzymatic grooves that work together to produce a 16-carbon chain saturated FA, palmitate, from acetyl-coenzyme A (CoA) and malonyl-CoA in the presence of nicotinamide adenine dinucleotide phosphate hydrogen (NADPH) [22]. The produce of cytosolic CoA, one of the FASN substrates, was through the action of ACLY [23]. ACACA, the rate-limiting enzyme for the long-chain FA synthesis that catalyzes the ATP-dependent carboxylation of acetyl-CoA to malonyl-CoA (also a FASN substrate) [24]. THRSP and its homolog Spot-related protein (MIG12, Spot14-R) can form heterodimers to modulate FA synthesis [25]. Our previous research observed that the lipid metabolism pathway, such as FA metabolism pathway was down-regulated in the 45-Gy electron beam irradiated mouses’ skin tissue [26]. Interestingly, in this study, we found that FA biosynthesis pathway was significantly up-regulated in radon-exposed skin (Fig. 3F).

miRNAs have been identified as promising biomarkers in cancer and other diseases. Past research has documented that mechanism underlying lung cancer induced by radon may involve alterations in the expression of miRNA-34a gene and consequent disturbances in apoptosis [27]. In this study, using miRNA sequencing we found that 29 miRNAs were over- and 28 were under-expressed in radon-exposed skin tissues, compared with nonirradiated tissues. The dysregulated miRNAs include miR-31-3p, miR-1247-5p, miR-378a-3p, miR-206-3p and so on. It has previously been reported that miR-206-3p was down-regulated by acute ultraviolet radiation in mice [28]. Dysregulated miR-206 expression has been implicated in multiple kinds of cancers, including breast, lung, colorectal, ovarian and prostate cancers [29]. In addition, recent studies have suggested that miR-378a-3p is significantly up-regulated in irradiated small intestine cells and tissues. miR-378a-3p overexpression represses the radiosensitivity of small intestine cells [30]. However, herein we found that miR-378a-3p was significantly down-regulated in radon-exposed skin tissues. miR-378a-3p decreases interleukin-33 levels and enhances the sensitivity of ovarian cancer cells to cisplatin through targeting MAPK1 and GRB2 [31, 32].

miRNAs exert their functions through mRNA decay or translation inhibition. In the latter mechanism, the mRNA level of a protein-coding gene usually remains unchanged, whereas protein level is affected. Here we analyzed the relationships between the miRNA and the protein profiles and identified that FASN is positively regulated by miR-378a-3p and miR-206-3p, which has not been reported previously. Based on our results, we postulate that miR-206-3p and miR-378a-3p may regulate FASN in indirect mechanisms, this needs to be further explored. FASN is responsible for the de novo synthesis of FAs, which is involved in the preservation of biological membrane structure, energy storage and assembly of factors involved in signal transduction [33]. In normal conditions, FASN converts excess carbohydrate into FAs that are then esterified to storage triacylglycerols, which, when necessary, provide energy through β-oxidation [20]. FASN not only integrate several signaling pathways that regulate the metabolism, proliferation and survival in tumor cells, but also play an active part in the development, maintenance and metastatic progression of human cancers [20, 21, 34, 35]. It has also been reported that diallyl sulfide (DAS) mediated modulation of FASN leads to cancer cell death in Benzoapyrene-induced lung carcinogenesis. DAS-induced apoptosis is strongly associated with the down-regulation of FASN in tumor tissues [36]. However, FASN contributes to restimulation-induced cell death of Human CD4 T cells [37]. This study indicated that overexpression of FASN in WS1 inhibit the proliferation, destroy the nucleus morphology and aggravate the apoptosis, especially late apoptosis. Based on the above research results, we speculate that radon exposure increased the expression of FASN in skin tissues. The increased FASN inhibited cell proliferation, increased cell deaths and ultimately inducing skin damage. FASN might function as a previously unrecognized metabolic intermediate of oncogenesis linking energy, anabolism and malignant transformation. FASN plays an important role in the occurrence of skin cancer. However, much additional work will be required to fully understand the regulatory actions that could emanate from FASN activity in normal and tumor cells.

## CONCLUSION

This study provides evidence that radon exposure destroys skin integrity, which is accompanied by dysregulated proteins and miRNAs expression. Specifically, FASN was positively regulated by miR-206-3p and miR-378a-3p, and involved in the pathogenesis of radon-induced skin damage. Our present findings illustrate the molecular changes during radon-induced skin injury and the potential role of FASN in inducing this damage.

## CONFLICT OF INTEREST

The authors state no conflict of interest.

## Supplementary Material

Supplementary_Table_rrac037
