# Supplementary material for: Proteomic and miRNA profiling of radon-induced skin damage in mice: FASN regulated by miRNAs
Source: J Radiat Res. 2022 Jul 5;63(5):706–18. doi: 10.1093/jrr/rrac037 (PMC9494515; doi:10.1093/jrr/rrac037)
Supplement: Supplementary_Table_rrac037 [file supplementary_table_rrac037.docx]

**Supplementary Table 1 miRNA sequences**

| **Name** | **Sequence** |
| --- | --- |
| Mimics negative control | 5’-UUCUCCGAACGUGUCACGUTT -3’ |
| Inhibitor negative control | 5’-CAGUACUUUUGUGUAGUACAA-3’ |
| miR-206-3p mimics | 5’-UGGAAUGUAAGGAAGUGUGUGGACACACUUCCUUACAUUCCAUU -3’ |
| miR-206-3p Inhibitor | 5’-CCACACACUUCCUUACAUUCCA-3’ |
| miR-378a-3p mimics | 5’-ACUGGACUUGGAGUCAGAAGGUUCUGACUCCAAGUCCAGUUU-3’ |
| miR-378a-3p Inhibitor | 5’-CCUUCUGACUCCAAGUCCAGU-3’ |
